# Supplementary material for: A Critical View of the Application of the APEX Software (Aqueous Photochemistry of Environmentally-Occurring Xenobiotics) to Predict Photoreaction Kinetics in Surface Freshwaters
Source: Molecules. 2019 Dec 18;25(1):9. doi: 10.3390/molecules25010009 (PMC7017383; doi:10.3390/molecules25010009)
Supplement: Supplementary file 1 [file molecules-25-00009-s001.zip › 3.2.4_gcc-4.4.0.zip!/3.2.4_gcc-4.4.0/3.2.4_gcc-4.4.0/doc/octave/html/interpreter/Terminal-Input.html]

Terminal Input - Untitled


Next: Simple File I/O,
Previous: Terminal Output,
Up: Basic Input and Output


---

#### 14.1.2 Terminal Input

Octave has three functions that make it easy to prompt users for
input. The `input` and `menu` functions are normally
used for managing an interactive dialog with a user, and the
`keyboard` function is normally used for doing simple debugging.

— Built-in Function: **input** (prompt)  
— Built-in Function: **input** (prompt, "s")  
> Print a prompt and wait for user input. For example,
>
> ```
>           input ("Pick a number, any number! ")
> ```
>
> prints the prompt
>
> ```
>           Pick a number, any number!
> ```
>
> and waits for the user to enter a value. The string entered by the user
> is evaluated as an expression, so it may be a literal constant, a
> variable name, or any other valid expression.
>
> Currently, `input` only returns one value, regardless of the number
> of values produced by the evaluation of the expression.
>
> If you are only interested in getting a literal string value, you can
> call `input` with the character string `"s"` as the second
> argument. This tells Octave to return the string entered by the user
> directly, without evaluating it first.
>
> Because there may be output waiting to be displayed by the pager, it is
> a good idea to always call `fflush (stdout)` before calling
> `input`. This will ensure that all pending output is written to
> the screen before your prompt. See Input and Output.

— Function File: **menu** (title, opt1, ...)  
> Print a title string followed by a series of options. Each option will
> be printed along with a number. The return value is the number of the
> option selected by the user. This function is useful for interactive
> programs. There is no limit to the number of options that may be passed
> in, but it may be confusing to present more than will fit easily on one
> screen.
>
>
>
> **See also:** disp, printf, input.

— Built-in Function: **yes\_or\_no** (prompt)  
> Ask the user a yes-or-no question. Return 1 if the answer is yes.
> Takes one argument, which is the string to display to ask the
> question. It should end in a space; yes-or-no-p adds
> (yes or no)  to it. The user must confirm the answer with
> RET and can edit it until it has been confirmed.

For `input`, the normal command line history and editing functions
are available at the prompt.

Octave also has a function that makes it possible to get a single
character from the keyboard without requiring the user to type a
carriage return.

— Built-in Function: **kbhit** ()  
> Read a single keystroke from the keyboard. If called with one
> argument, don't wait for a keypress. For example,
>
> ```
>           x = kbhit ();
> ```
>
> will set x to the next character typed at the keyboard as soon as
> it is typed.
>
> ```
>           x = kbhit (1);
> ```
>
> identical to the above example, but don't wait for a keypress,
> returning the empty string if no key is available.
